# The Role of Molecular Investigations in Estimating the Time since Deposition (TSD) of Bloodstains: A Systematic Review of the Literature

**DOI:** 10.3390/ijms25137469

**Published:** 2024-07-08

**Authors:** Matteo Antonio Sacco, Saverio Gualtieri, Alessandro Pasquale Tarallo, Luca Calanna, Raffaele La Russa, Isabella Aquila

**Affiliations:** 1 Institute of Legal Medicine, Department of Medical and Surgical Sciences, “Magna Graecia” University, 88100 Catanzaro, Italy; matteoantoniosacco@gmail.com (M.A.S.); saveriogualtieri@icloud.com (S.G.); drtarallomedlegale@gmail.com (A.P.T.); luca.calanna@studenti.unicz.it (L.C.); 2 Department of Clinical Medicine, Public Health, Life Sciences, and Environmental Sciences, University of L’Aquila, 67100 L’Aquila, Italy; raffaele.larussa@univaq.it

**Keywords:** time since deposition, forensic sciences, bloodstains

## Abstract

At many crime scenes, investigators are able to trace and find traces of blood. For many years, it was believed that such traces could only be subjected to genetic investigations, such as those aimed at comparing DNA profiling with a suspect to verify his identity, and that it was therefore not possible to backdate the traces. In recent years, various works have used experimental models to investigate the possibility of identifying markers and methodologies for estimating the time since deposition (TSD) of bloodstains. Despite the results, these methods are still not part of standard procedures, and there is no univocal analysis methodology. In this work we carried out a systematic literature review of all the papers published in the last ten years on this topic, comparing the experimental models created. This review demonstrates the potential that different molecular approaches, such as transcriptomics, metabolomics, proteomics, and spectrometry, can have in the analysis of TSD, with notable sensitivity and specificity. This paper also analyzes the intrinsic and extrinsic limits of these models and emphasizes the need to continue research work on this topic, considering the importance that this parameter can assume in forensic investigations against a suspect.

## 1. Introduction

Blood deposition analysis serves as a cornerstone in the realm of forensic investigations, shedding light on the complex narratives of crime scenes. The significance of blood deposition analysis cannot be overstated, as it not only aids in reconstructing the sequence of events but also plays an important role in identifying the perpetrators and victims involved [1]. It plays a pivotal role in constructing a timeline of events, especially in criminal cases where the exact timing of an incident could lead to identifying or exonerating potential suspects. 

In particular, in some cases, especially when many days have passed since death, the reconstruction of the post-mortem interval (PMI) on the cadaver becomes more complex because of cadaveric transformative phenomena [2]. In other cases, during the inspection, the scene does not immediately point towards a homicide, and this diagnosis is therefore discovered only at autopsy. In these cases, the discovery of biological traces at the crime scene and, above all, a reliable backdating of these traces can make a useful contribution to defining the time of crime occurrence [3]. For many years, it was believed that it was not possible to backdate the deposition time of blood traces found at a crime scene but only to perform a genetic analysis [3]. In recent years, the scientific literature has begun to demonstrate that it is possible to backdate a trace by exploiting the advanced molecular methodologies available. In addition, forensic molecular biology has introduced an innovative concept known as trace deposition timing, which aims to estimate the timeframe of blood deposition with remarkable accuracy [4,5,6]. These advancements have the potential to provide investigators with precise timelines of criminal events, significantly impacting the outcome of forensic investigations [7,8].

The impact of research on the TSD of bloodstains in forensic sciences can be significant. In crime investigations, correct backdating could allow us to understand the time when a crime occurred and the recurrence of violence in different eras. Knowledge of this information should be combined with estimating the PMI, allowing us to improve its precision and verify its reliability. Furthermore, the estimation of the TSD of bloodstains could replace PMI evaluation when it is not possible to perform reliable backdating because of the transformative phenomena of a cadaver. Finally, since in a violent situation, often not only the victim but also the attacker may lose blood and leave traces at the time of the fight, estimating the TSD, combined with genetic investigations, could prove decisive in demonstrating the presence of the attacker at that moment. Despite the advancement of scientific research in this area, to date, these procedures are not part of standard protocols or methodologies used in forensic practice. For this reason, despite the potential application of many of these techniques, these systems are still not well known or applied for judicial purposes. These analyses have the potential to be applied at the scene, similar to the application of forensic genetics, but this requires the analysis of a greater number of experimental models and a procedural uniformity that has not yet been achieved with the creation of statistically significant evidence.

The aim of this work is to identify the state of the art of TSD estimation, describing the advances in molecular biology and its practical applications at crime scenes. To this end, this paper traces all the experiments that have been carried out by researchers in the last 10 years. Furthermore, we discuss the technical limitations of these investigations and future research prospects. This work represents, to our knowledge, the first review of the literature on this topic.

## 2. Materials and Methods

A systematic review of the literature was carried out using the PubMed NCBI and Scopus search engines. The search was performed by entering the following Mesh Terms: blood deposition time and forensic. Only English papers published in the last ten years (2014–2024) were included in the search. We included papers that performed experiments with molecular biology methods or innovative technologies. In particular, papers that analyzed experimental laboratory models reproducing multiple bloodstains at predetermined chronological intervals were included. Papers analyzing case reports were also included. Works that dealt exclusively with the evaluation of trace conservation methodologies in extreme environmental conditions but did not estimate TSD were excluded. Furthermore, works that did not aim to evaluate TSD but other variables such as the sex or age of the victim were excluded. Also, literature reviews were excluded. This research was carried out by two independent operators according to the PRISMA Guidelines and the results were subsequently compared [9]. The works were initially selected based on titles after the removal of duplicates. Subsequently, the abstract was read, and the works whose full texts were available were selected. A risk of bias assessment was performed according to the Joanna Briggs Institute Critical Appraisal Checklists [10].

## 3. Results 

### 3.1. Number of Papers

**Figure 1:**
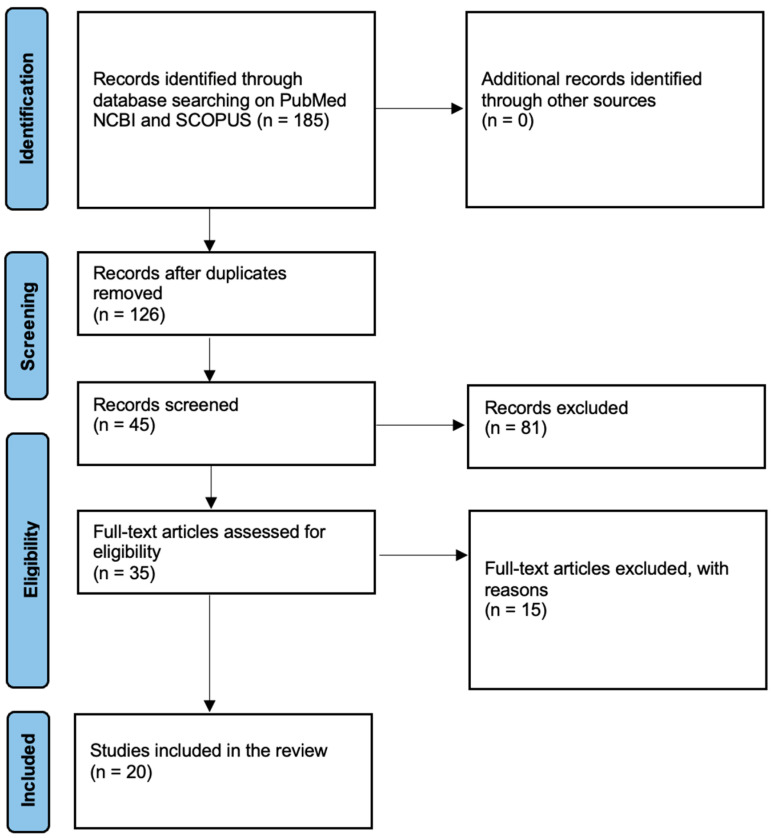
Searching method using the PRISMA flowchart.

The search found a total of 185 titles by entering the keywords in the two search engines. After removing duplicates, 126 papers were obtained. Subsequently, 45 abstracts, published in the last 10 years, were selected for reading. Of these, 35 papers were then read in full, and a total of 20 papers that corresponded to our inclusion criteria were finally selected (Figure 1). In total, 15 articles were excluded as they did not provide information on how to estimate TSD but analyzed other variables such as sex, age, or environmental conditions on trace degradation. Our results show a strong increase in research work in the last 10 years compared with the previous decade. In particular, a large number of these works were published in the last 3 years (50%).

### 3.2. Type of Experimental Model

In almost all cases, the experimental models were carried out in the laboratory with human blood taken from voluntary subjects (95%). In only two papers, the research was carried out on mouse models (10%).

### 3.3. Molecular Targets

Most studies focused on RNA research (40%). In particular, the authors examined the degradation pattern, mechanisms of up- and downregulation of mRNA, and quantification of circRNA expression levels as a function of elapsed time. In 30% of the papers, the target of the investigations were proteins with the choice of various markers including hemoglobin or its subunits, plasma metabolites with the selection of candidates according to metabolomics approaches, and alkaline phosphatase. In other works (10%), the authors evaluated fluorescence levels related to changes over time determined by tryptophan, nicotinamide adenine dinucleotide (NADH), and flavins.

In one work (5%), the surface characteristics of the bloodstains were evaluated using an optical profiling approach, and in another (5%), the absorbance levels were analyzed. In one work (5%), STR profiles were analyzed on three different fabrics.

### 3.4. Experimental Analyses

The experimental models involved taking blood from volunteer subjects or from animals. The samples collected were peripheral blood or menstrual blood. The deposition strategies were different including in vitro analysis, on cotton stubs, or placing traces on different tissues or on special cards.

In the cases that analyzed mRNA or DNA, the investigations were carried out using quantitative PCR analysis or RNA sequencing (45%). DNA analysis occurred through extraction and quantification. In the case of RNA analysis, the authors provided RNA extraction with quality assessment, library preparation and sequencing according to manufacturer instructions, RNA sequencing, and statistical analysis. In the other works, the investigations were carried out with immunochromatographic tests (5%), LC/MS methods (20%), spectrophotometric methods (5%), and spectroscopy methods (20%). Among the other investigations, optical profilometry (5%) was highlighted.

For immunofluorescence investigations, special commercial kits were used with band intensity scale analysis. In LC/MS analyses, the authors completed sample preparation on a fraction of a bloodstain, which was processed in an Eppendorf tube with the use of extraction buffer, followed by vortexing and incubation in an ultrasonic bath, precipitation, centrifugation, and drying the supernatant using gentle nitrogen. Then, chromatographic separation with gradient elution and mass spectrometric detection were performed. For spectroscopy, various strategies such as fluorescence spectroscopy, Raman, and ATR-FTIR were performed.

For optical profilometry, the instrument was used to scan the bloodstains with a 20x objective lens, and surface average roughness, root mean square roughness, kurtosis, and skewness were assessed.

### 3.5. Ages of Bloodstains

Analysis times ranged from 0 to a maximum of 1.5 years. In particular, the majority of the works (45%) analyzed an interval of hours (0–168 h). Other works (15%) examined a timescale of up to 30 days. Overall, 40% of the papers evaluated a time greater than 30 days up to a maximum of 1.5 years (Table 1).

## 4. Discussion

**Figure 2:**
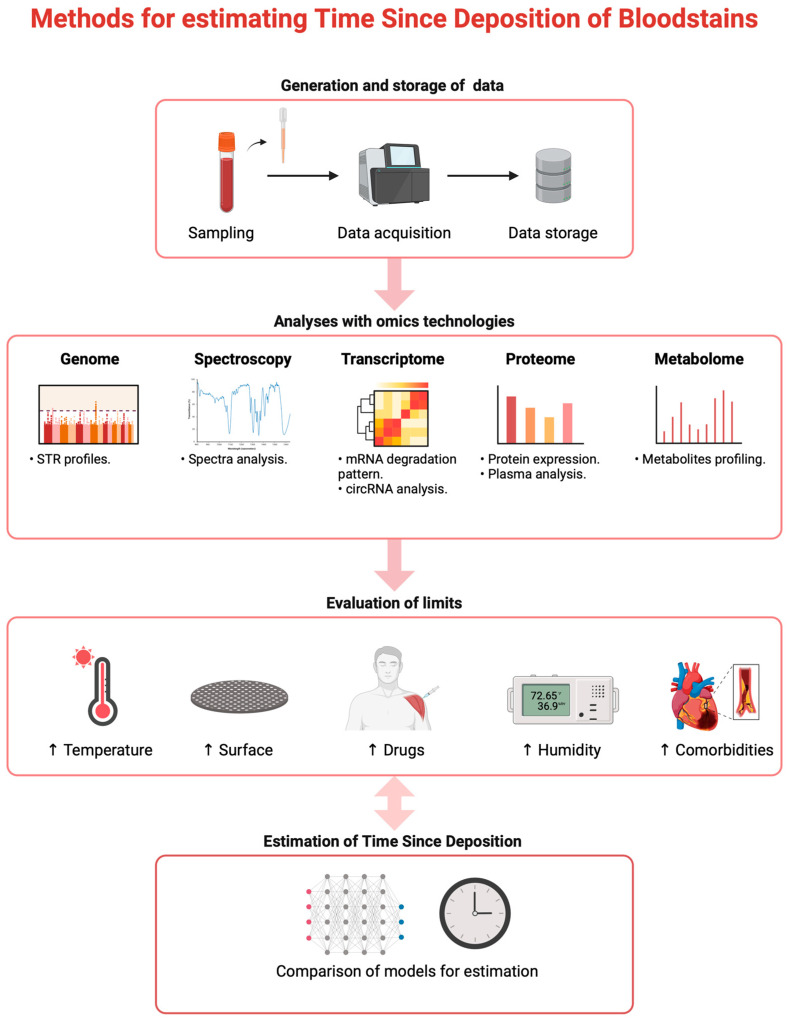
Methods for estimating trace deposition time and limits.

Methods for estimating the age of bloodstains at a crime scene have evolved significantly, incorporating both traditional and advanced technological approaches [12,13,14,15,16,17] (Figure 2). In particular, this review demonstrates how genetics and, therefore, DNA analysis can identify the donor profile but offer little prospect with respect to TSD. Genetic data, although very useful, actually has limitations in investigations considering that the DNA profile identified must always be compared with another DNA profile of a suspect to be useful. It should also be taken into account that the suspect and the victim could have had more than one encounter (as happens in repeated abuse), so the blood traces found at the scene could refer to different moments in time and, therefore, to more than one episode [30,31,32]. In these cases, it is essential to adopt techniques that allow for the backdating of traces.

This review demonstrated the applicability of a series of molecular targets in the estimation of TSD. Most of the works evidenced, especially in recent years, the usefulness of studying transcriptomics. Although mRNAs are known to be unstable, several studies have evaluated their stability and applicability in studying trace amounts of biological fluids. Furthermore, the degradation pattern that characterizes mRNAs can prove valuable in the analysis of TSD. Zhang et al. recently built a random forest prediction model that allowed the identification of a total of 11 upregulated transcripts and 13 downregulated transcripts [11]. With a similar model, Gosch et al. analyzed a series of eight time intervals on the day of the bloodstains, evaluating 13 candidate markers for the estimate [12]. Hänggi et al. focused their experimental model on four transcripts (B2M, LGALS2, CLC, and S100A12) by targeting the 5′ and 3′ ends of the marker Aminolevulinate synthetase 2 (ALAS2) [11].

Furthermore, recently, the expression levels of circRNAs have also been evaluated because of their greater stability [17]. Other authors have considered the level of rhythmic mRNA with the construction of machine learning models aimed at predicting TSD over a 24-h period [24,29]. Using similar approaches, Salzmann et al. evaluated the applicability of the RNA degradation pattern by comparing indoor and outdoor environments and noted, in indoor conditions, the possibility of evaluating degradation mechanisms for up to one and a half years, but with limits related to the environmental factors that make RNA more unstable [22].

One pioneering method involves the use of Raman spectroscopy, which has shown promising results in determining the age of bloodstains up to two years. This technique assesses changes in the molecular composition of blood, providing an estimate of its degradation over time. Additionally, the application of electrochemical methods to study degrading bloodstains offers a new avenue for estimating TSD by assessing hemoglobin-related measurements. A large number of works have considered the impact of spectrometry in their analyses. Fonseca et al. created a stain classification model with hierarchical modeling and a handheld NIR spectrometer, capable of evaluating stains between 0 and 30 days with good sensitivity and specificity [25]. Wójtowicz et al. considered a model based on fluorescence spectroscopy that highlights changes in the first 9 h. These findings were attributed to changes in tryptophan, nicotinamide adenine dinucleotide (NADH), and flavins over time [23]. Agudelo et al. also evaluated the use of spectrophotometry to investigate the marker Alkaline phosphatase (ALP) with catalytic assay. Spectrophotometric methods were also used with colorimetric methodology [28].

Schneider et al. investigated the utility of identification-based LC-MS approaches using bottom-up proteomics by identifying the usefulness of some peptides and amino acids for the estimation [18]. The same author also investigated the role of the dipeptide Phenylalanylalanine (PheAla) using LC-HR-MS. Lech et al. evaluated a predictive model over a 36-h period starting from 171 plasma metabolites and arriving at 10 protein biomarkers [25]. Among other proteins, we must also consider the role of hemoglobin. Hemoglobin variations were successfully investigated in a model created by Heo et al., who investigated changes in the beta subunit over time. These variations were also investigated in a recent model created by Medina et al., who analyzed, using immunochromatographic tests, this protein by comparing different surfaces [3,19].

The impact of environmental conditions on the degradation of bloodstains significantly complicates the estimation of TSD [33,34]. Research has demonstrated that peripheral bloodstains degrade at a faster rate when exposed to extreme thermal environments compared with those aged under ambient conditions, indicating the substantial influence of temperature on blood degradation rates [35]. Moreover, environmental factors such as humidity, exposure to sunlight, and the presence of microorganisms can further accelerate the degradation process, affecting the accuracy of TSD estimations. These findings emphasize the critical role of environmental conditions in the forensic analysis of bloodstains, highlighting the importance of considering these factors when attempting to determine the time since deposition of blood at crime scenes [36].

Among the limitations of these analyses, we must also take into account the potential interindividual variability related to parameters such as sex, age, comorbidities, and previous pharmacological treatments (such as anticoagulants) in the evaluation of molecular targets [37,38]. Regarding sex and age, very few studies have evaluated the influence of these variables on estimates of TSD. Recently, in an experimental metabolomics study in which adenosine 5′-monophosphate, choline, and pyroglutamic acid were selected as markers, Lee et al. identified higher levels of seven metabolites in women at time 0 compared to men [31]. Agudelo et al. evaluated the levels of alkaline phosphatase (as they are related to the age of the subject) by dividing traces into two groups, i.e., young and old originators, and demonstrated that its levels were different in the groups. Therefore, the influence of interindividual variability is possible but depends on the type of marker selected for the investigations [28]. Furthermore, a blood sample is not always of sufficient quantity or quality to allow for an analysis with a high degree of sensitivity and specificity or to allow for repeatability of the investigation. Therefore, it is necessary to consider the possibility of dating experimental models on numerically very small samples, which would then require transposition onto larger models. Also, we reiterate the role of extrinsic environmental factors in the degradation of traces, making it necessary to compare indoor and outdoor experimental models. Also, a decisive role is played by the type and color of the surface on which the trace is found (floor, fabric, etc.); therefore, a comparison among different surfaces is also necessary and has already been carried out in some works [3]. The surface type can significantly influence the speed and intensity of drying and absorption of a stain, especially textiles. Among the limitations, we consider that various types of pre-treatments have been used in the literature. For example, some authors pre-treated a sample with EDTA, while others did not apply any anticoagulant. We consider that since the use of anti-coagulants affects the characteristics of blood and its fluidity, the analysis strategy should take this variable into account by creating models with and without pre-treatment with anticoagulants. Furthermore, the methods and dimensions by which blood traces were obtained and analyzed were different. Therefore, the creation of an experimental model should always consider the same size of stains and the same volume of blood. Finally, various timescales were used by the authors, both short- and long-term, up to a maximum of 1.5 years, according to individual selection criteria.

### Future Directions

In the coming years, the backdating of bloodstains at crime scenes will aim to become increasingly precise. To this end, future research works should evaluate experimental models with uniform methods considering the intrinsic and extrinsic variables described. The implementation of molecular investigations also has economic aspects that should be considered due to the use of instruments, purchase of reagents, and human resources for data analysis and interpretation. Therefore, the research and application of economic methods would be desirable considering that multiple traces may need to be analyzed. From this perspective, these investigations could be performed in all centers, not just in referral centers. From a logistical point of view, particular attention should be paid to the correct chain of custody of biological evidence before analysis, especially in the transport phase from the crime scene to the laboratory, and the potential risk of contamination. Also, investigators should consider the quantity of biological material available for analysis and select a strategy that allows the use of the smallest quantity of trace and the repetition of the assessment for judicial purposes.

## 5. Conclusions

Despite the challenges associated with these methods, their successful application in the works described confirms the potential of molecular investigations in the TSD of blood traces. In this context, we emphasize the need to increase the number of experimental models created and, above all, the need to make these models applicable to reality by introducing them into forensic practice. In this regard, we highlight the potential of artificial intelligence in the generation of algorithms that help produce predictive models with respect to the results and increase work in a similar way to what is already happening in the forensic field of PMI research. 

## Figures and Tables

**Table 1:** Table of study characteristics.

Authors	Year	Methods	Molecular Target	Name of Target	Time Analyzed	Sample	Environmental Condition	Result
Medina-Paz et al. [3]	2024	Immunochromatographic tests and qPCR	Protein and DNA	Hemoglobin STR profiles	1–90 days	Human (n = 1)	Three different fabrics including denim (dyed black and white), cotton (dyed black and white), and natural leather at room temperature	It was possible to recover and identify blood samples up to three months after deposition and to obtain full STR profiles from all the tested fabrics. STR profiles showed differences in their quality between 1 and 90 days after deposition for all fabrics.
Zhang et al. [11]	2024	RNA sequencing	mRNA	Transcriptome	0–168 days	Humans (n = 20)	In vitro at 4 °C	Eleven upregulated and thirteen downregulated transcripts were identified as potential time-marker transcripts.
Gosch, et al. [12]	2023	Quantitative PCR analysis	mRNA	Eighty-one selected mRNA markers	0–24 h	Human (n = 10)	-	It was possible to use 13 selected candidate markers for the estimation.
Hänggi et al. [13]	2023	Quantitative PCR analysis	mRNA	Four transcripts (B2M, LGALS2, CLC, and S100A12)	1–2 h after deposition, 310 days	Human (n = 5)	Room temperature and exposure to some artificial light during daytime	B2M, S100A12, and CLC showed time-dependent RNA degradation patterns.
Cheng et al. [14]	2023	Quantitative PCR analysis with machine learning	mRNA	Seven transcripts (STAT3, TRIB1, CDKN1A, PER1, MAP kinase, MKNK2, THRA, SREBF1)	24 h	Human (n = 8)	-	mRNA biomarkers could be used to estimate the bloodstain deposition time within a 24-h period.
Vale et al. [15]	2023	Optical profilometry	Surface characteristics	Surface average roughness, kurtosis, skewness, maximum height, number of cracks and pits, and height distributions	0–4 weeks	Animal (bovine)	Room temperature	The majority of the changes in surface characteristics occurred in the first 35 min after bloodstain deposition, in agreement with current research in bloodstain drying.
Schneider et al. [16]	2023	LC-HR-MS	Protein	Metabolome	48 weeks	Human (n = 11)	Samples were divided in indoor and outdoor	The dipeptide Phenylalanylalanine (PheAla) showed strong association for TsD prediction.
Wei et al. [17]	2022	Real-time quantitative polymerase chain reaction (qPCR)	circRNAs and mRNAs	mRNA markers (GYPA, CD93, ALAS2, SPTB, HBB, HBA), expressed circRNAs in human peripheral blood (hsa_circ_0001445, hsa_circ_0000972, hsa_circ_0000095) and reference genes (18 S, ACTB and U6)	0–120 days	Human (n = 10)	Samples were divided in indoor and outdoor	The expression levels of hsa_circ_0001445, ALAS2, and HBB could be used to estimate the TsD of bloodstains.
Schneider et al. [18]	2022	HR-LC-MS	Proteome	-	2 months	-		Certain peptides and amino acid modifications were identified and further assessed for their applicability in assessing passed TsD. A prediction model based on data resampling (Jackknife) was applied.
Heo, et al. [19]	2022	LC-MS	Protein	Hemoglobin subunit beta	0–30 days	Human (n = not reported)	Dried and humidity of 30%, 60%, and 90% at room temperature	Hemoglobin subunit beta protein levels showed a gradually increasing pattern. Significant differences were found among the samples.
Marrone et al. [20]	2021	Colorimetric methodology with a spectrophotometer	Color	-	24 h/60 days	Human (n = 8)	An air-conditioned room at 25 °C during the day	The authors developed two bloodstain age prediction algorithms including a short-term and a long-term useful model for the first 24h and 60 days, respectively. Both models showed high levels of classification accuracy, particularly for the long-term model.
Weber et al. [21]	2021	Fluorescence spectroscopy	Fluorescence	Tryptophan, nicotinamide adenine dinucleotide (NADH), and flavins	1–24 h	Human (n = 2)	Not reported	Fluorescence of bloodstain changed significantly during 24 h post deposition.
Salzmann et al. [22]	2021	Quantitative PCR analysis	RNA degradation pattern	-	1.5 years	Human (n = 3)	Indoor (dark, room temperature) and outdoor (exposed to sun, wind, etc., but protected from rain)	RNA degradation patterns were identified with several candidate markers. The indoor samples showed a marked drop in RNA integrity after 6 months, while the outdoor samples were difficult to interpret.
Wójtowicz et al. [23]	2021	Fluorescence spectroscopy	Fluorescence	Tryptophan, nicotinamide adenine dinucleotide (NADH), and flavins	1–9 h	Human (n = 2)	Room conditions	Steady-state fluorescence spectra underwent significant changes over the first nine hours post-deposition.
Asaghiar et al. [24]	2020	Quantitative PCR analysis	mRNA	Vascular Endothelial Growth Factor A (VEGFA) and Hypoxia-Inducible Factor 1 Alpha (HIF1A)	0–28 days	Human (n = 8)	Room temperature	A stain age prediction model based upon VEGFA with ACTB as a reference gene could be used on samples up to four weeks old.
Lech et al. [25]	2018	LC/MS	Proteins	One hundred seventy-one plasma metabolites	0–36 h	Human (n = 8)	Not reported	The prediction model established here utilized 10 metabolite biomarkers for estimating three day/nighttime categories
Zhang et al. [26]	2017	Attenuated total reflection (ATR) Fourier transform infrared (FTIR) spectroscopy	Absorbance	Absorbance at 3308/cm (A3308) and 1541/cm (A1541)	Relatively early period (from 0 min to the time required for the bloodstain to dry out)	Rat (n = 80) Human (n = 10)	Room temperature	The absorbance at 3308/cm (A3308) was found to have a close correlation with TSD during the time period, and the changes in A3308 during the drying of rat and human blood drops under the same controlled conditions showed similar results.
Doty et al. [27]	2016	Raman spectroscopy	Protein	Hemoglobin	1–168 h	Human (n = 2)	Blood on aluminum foil at room temperature	There was a high correlation between several Raman bands and the age of a bloodstain on the scale of hours to days.
Agudelo et al. [28]	2016	Spectrophotometry	Protein	Alkaline phosphatase (ALP)	0–48 h	Human (n = not reported)	Room temperature	The catalytic essay of ALP can be used for Simultaneous Estimation of the Time since Deposition and Age of Its Originator
Lech et al. [29]	2016	Quantitative PCR analysis	mRNA	Twenty-one candidate mRNA markers	2–36 h	Human (n = 12)	Not reported	Three mRNA markers, HSPA1B, MKNK2, and PER3v melatonin, and cortisol allowed for estimating three time categories for TDB.

## Data Availability

Not applicable to this article as no datasets were generated.
